# Cross-Cultural Awareness and Attitudes Toward Threatened Animal Species

**DOI:** 10.3389/fpsyg.2022.898503

**Published:** 2022-05-31

**Authors:** Jennifer Bruder, Lauren M. Burakowski, Taeyong Park, Reem Al-Haddad, Sara Al-Hemaidi, Amal Al-Korbi, Almayasa Al-Naimi

**Affiliations:** Department of Arts and Sciences, Carnegie Mellon University in Qatar, Doha, Qatar

**Keywords:** culture, animal, attitudes, liking, familiarity, conservation, Qatar, Arabian Gulf

## Abstract

The preservation of our planet’s decreasing biodiversity is a global challenge. Human attitudes and preferences toward animals have profound impacts on conservation policies and decisions. To date, the vast majority of studies about human attitudes and concern toward animals have focused largely on *western, educated, industrialized, rich* and *democratic* (i.e., WEIRD) populations. In order to mitigate biodiversity loss globally, an understanding of how humans make decisions about animals from multicultural perspectives is needed. The present study examines familiarity, liking and endorsement of government protection amongst six broad cultural groups living in Qatar for five threatened animal species indigenous to the Arabian Gulf. Our findings highlight similarities and differences across cultures toward animals. Overall, familiarity did not predict endorsement for government protection after liking was accounted for. Liking, however, emerged as an important predictor of endorsement for government protection across cultures, although the degree of animal liking varied culturally. WEIRD and South East Asian participants showed similar and more positive attitudes toward animals compared to the other groups. Participants from the Arabian Gulf, Sub-Saharan Africa, Middle East and North Africa, and South Asia responded similarly toward the animals. Interestingly, the Arabian Gulf group demonstrated significantly less liking and protection endorsement for animals, including those animals which play an important role in their culture. This research highlights intriguing avenues for future research and points to liking as a possible universal human attitude toward animals that influences decision making about conservation across all cultures while suggesting applications for improving education.

*On aime ce qui nous a émerveillé, et on protège ce que l’on aime. - Jacques Cousteau*

*In the end we will conserve only what we love. We will love only what we understand. We will understand only what we are taught. - Baba Dioum*

## Introduction

The rapid loss of biodiversity on our planet is a global challenge. According to the International Union for Conservation of Nature Red List (IUCN), the number of animals worldwide that are added to endangered and critically endangered lists is rapidly increasing (International Union for Conservation of Nature, 2021). Biodiversity loss is overwhelmingly accelerated by humans, who are altering the planet at an unprecedented rate (Steg and Vlek, 2009; Secretariat of the Convention on Biological Diversity, 2020; WWF, 2020).

Reversing biodiversity loss requires a holistic and representative understanding of human attitudes and behavior toward animals across all cultural perspectives. Because animal conservation is a global issue, it is necessary to understand human behavior collectively. However, investigations of human attitudes toward animals have been predominantly conducted with WEIRD (*western, educated, industrialized*, *rich, democratic*) populations (e.g., Amiot and Bastian, 2014), or have explored attitudes toward animals in isolated cultural contexts with regionally-specific questions (e.g., Heinen and Shrivastava, 2009; McLennan and Hill, 2012; Buckley et al., 2017). Research on the influence of human attitudes toward animals and effects on policy clearly demonstrate how human preferences for certain types of animals influence decision making and policies related to conservation efforts (Coursey, 1994; Metrick and Weitzman, 1996; Czech et al., 1998; Knegtering et al., 2002; Tisdell et al., 2005; Mahoney, 2009; Tisdell, 2014); however, these studies are limited in their global reach. Thus, scientists, policymakers and educators need to understand how all people decide the worth of animals. This understanding can be leveraged to improve people’s perception of animals and in turn to nudge behavior and decision making in support of biodiversity initiatives. More concretely, this knowledge can aid in the development of conservation programs for global audiences. One promising avenue of research is to determine if there are psychological universals related to human attitudes toward animals that might be leveraged to empower global policymakers, educators and scientists to create overarching global mitigation strategies (i.e., Norenzayan and Heine, 2005). To empower these stakeholders, cross-cultural research needs to explore important questions including how humans decide the worth of animals, how these decisions relate to perceived need for protection for animals, and how these decisions influence human behavior and government policy.

One method researchers have employed to investigate the relationship between attitudes toward conservation and biodiversity is the willingness-to-pay (WTP) procedure (Martín-López et al., 2007; Tsi et al., 2008; Wang and Jia, 2012; Echeverri et al., 2017; Dörge et al., 2022). The WTP procedure when used to study attitudes toward animals assumes that people will pay for the conservation of an animal according to their perceptions of that animal’s worth, typically in the form of a monetary donation to support conservation. The definition of worth is largely subjective, and may include many criteria such as aesthetic, emotional, economic and scientific factors. This paradigm allows researchers to explore which factors motivate decision making around individual conservation behaviors.

Using intended donations as a measure for conservation intentions in the general population, Echeverri et al. (2017) asked Canadian undergraduates how much they liked, were familiar with and would be willing to donate to conserve four endangered North American animals. Participants’ subjective familiarity with an animal correlated positively with liking for each animal, suggesting that the more subjects felt they knew about a particular animal, the more they liked them. Familiarity was found to be unrelated to one’s intention to donate for conservation, whereas liking emerged as a significant predictor of participants’ WTP for an animal’s conservation. These results suggest that although knowledge leads to more positive attitudes toward animals, it is the positive attitude, not the knowledge, that results in pro-conservation intentions toward animals. Beliefs about endangered status only influenced intended donations for the most liked and least liked animals in the study. In a follow-up study (*Ibid*), the researchers asked American and Indian M-Turk participants about their familiarity, liking and WTP for the same animals. Once again, subjective familiarity was positively related to liking. Liking again predicted intended donations for all animals; however, familiarity also emerged as a significant predictor of intended donations for three of the four animals. Thus, the role of liking and familiarity were not uniform across these two studies. One reason for this finding may be related to culture; however, the authors did not analyze the results of their study by culture, so it is not possible to know if culture played a role in the findings.

Martín-López et al. (2007) surveyed Spanish and European National Park visitors on animal preference and measured their WTP for 15 species native to the National Park (they also included 5 plant species). Participants differed on their motivations to visit the park, and these motivations were related to tendencies to think about animals from different perspectives. For example, short-term visitors tended to view animals on utility and familiarity, whereas nature enthusiasts and environmental professionals had stronger stances related to ethics and ecological-scientific considerations. All visitors ranked animals in a similar order, indicating strong positive attitudes toward megafauna, and ranked birds highest overall, followed by mammals, reptiles, fish, and invertebrates. Overwhelmingly, the authors found affective factors (i.e., animal liking), more strongly influenced WTP than economic factors or scientific considerations, especially amongst visitors with less knowledge and concern about environmental issues. Knowledge and education about biodiversity issues did moderate the results for visitors with expert knowledge about the animals, suggesting an important role for specific education on these issues.

In France, Colléony et al. (2017) measured actual donations as part of an animal adoption program given for endangered zoo animals, against the type of animal adopted, animal charisma, phylogenetic distance from humans, IUCN threat level and the order the animal appeared alphabetically in the adoption list. Animals with higher charisma ratings were more likely to be adopted, although they received less funding than non-charismatic animals, suggesting that those people who chose less charismatic animals more thoughtfully participated in the adoption program. Interestingly, adoption of a non-charismatic animal did not correlate with biodiversity concern. Additionally, animals that appeared at the beginning of the alphabetically ordered adoption list were also more likely to be adopted, suggesting participants employed a cognitive shortcut to make their adoption decision. Finally, the IUCN threat level of the animal had no influence on animal adoption. Similarly, in a survey of Slovak zoos, Fančovičová et al. (2021) found zoo donations to be influenced most strongly by animal attractiveness and phylogenetic closeness to humans, where threat status only played a small role compared to animal factors. These findings highlight the possibility that people take a simple and biased approach to animal conservation decisions, picking animals which are most liked and most readily available. It is important to underline potential differences between paradigms that measure intended versus actual donations to animal conservation. For example, in Germany Dörge et al. (2022) measured both intended and actual donations to insects. Though all respondents were favorable toward insect conservation, a significant intention-behavior gap was found in their response patterns, where their intentions to donate to insect conservation were larger than their actual donations. These findings suggest that using the willingness to pay procedure has some limitations when drawing conclusions about how people will actually behave (also see Kanya et al., 2019).

Results from studies employing the WTP procedure comparing different types of animals highlight how personal preferences toward animals have a stronger impact on decision making than knowledge about the animals or endangered status in the general population (for further discussion see: Gunnthorsdottir, 2001; Marešová and Frynta, 2008; however, for different perspectives see Tisdell, 2014). The notion that people are willing to pay more for certain types of animals is in line with research on flagship species (Clucas et al., 2008; Small, 2012). Flagship species are popular, charismatic animals that serve as symbols for acquiring public support to promote conservation awareness and action. Examples of flagship species include the giant panda (*Ailuropoda melanoleuca*), the tiger (*Panthera tigris*) or the African elephant (*Loxodonta africana*), all of which have subjective charm or a defining, appealing characteristic (Ducarme et al., 2013). Due to their perceived charisma, flagship species can generate increased conservation donations (e.g., Bowen-Jones and Entwistle, 2002; Fazey et al., 2005; Sitas et al., 2009), and play a crucial role in conservation programs, though this comes with both advantages and disadvantages (see Ducarme et al., 2013). Overall, charismatic species are typically large mammals and vertebrates who possess something people value as attractive and/or positive, such as intelligence, beauty, valor, singularity or strong symbolism.

Taking into consideration the role that human attitudes in western contexts play in conservation behavior and that these attitudes are moderated by preferences for animals, it is therefore important to understand to what degree, if any, human attitudes toward animals vary across cultures (Norenzayan and Heine, 2005). Arguably, animal charisma is subjective and differences in animal liking may be reasonably assumed to exist across cultures. Indeed, culture has been demonstrated to have important consequences for broader conservation attitudes, where interactions with religion, childhood experiences, diet, gender and age have been found to shape thought (e.g., Busch et al., 2020; Randler et al., 2021); however, even in broader contexts related to human-environment interactions, the literature lacks cross-cultural investigations (Tam and Milfont, 2020).

The present study aims to address these attitudes in a multi-cultural context in Qatar, a small, rich peninsular state in the Arabian Gulf. Qatar’s terrestrial habitat is arid desert and the coastline is long, shallow and warm. Compared to WEIRD countries, Qatar is arguably *Educated*, *Industrialized* and *Rich*, but it is neither *Western* nor *Democratic*. Its population is extraordinarily multicultural. Approximately 85% of Qatar’s population are expatriates. Qatar does not have an immigration policy, so foreigners living in Qatar stay only for the duration of their work visas (a few months to 20+ years). In part because there is limited long term immigration in the country, there is little mixing between citizens and expatriates, and people tend to maintain close ties to their home cultures and traditions. Estimated population demographics reveal the rich nature of the multi-cultural environment in Qatar: Indian nationals are the most prevalent group (22%), followed by Nepalese and Bangladeshi (13%), other Arab (13%), Filipino (7%), Pakistani (4%) and Sri Lankan (5%), and interestingly, Qatari nationals (11%) are a minority in their own country. The percentage of WEIRD foreigners is around 5%. The vast majority of people in Qatar (>90%) live in the capital city, Doha (Snoj, 2019).

We launched a nation-wide survey to query citizens and expatriates (residents) about their awareness and attitudes toward five threatened species indigenous to the Arabian Gulf region. The present study is unique in that it measured attitudes toward threatened species across a diverse range of cultures all living in the same geographical area, and compared these attitudes to the most commonly investigated WEIRD population. Many of the participants are citizens of countries which have not been previously studied with regard to awareness and attitudes toward animals and conservation. In order to understand basic awareness of the animals, we first asked participants to indicate their degree of familiarity with each animal, followed by whether or not they liked the animal. We then asked participants to which degree they endorsed the animal for government protection. We replaced willingness to pay with willingness to endorse government action in order to avoid potential cultural confounds related to perceptions and attitudes toward charity. Specifically, culture is a source of psychological and behavioral variation, and thus, the concept of charity may be culturally variable (e.g., Norenzayan and Heine, 2005). In addition, there is a wide disparity between income levels in Qatar, so the meaning of a donation amount will not be consistent across groups.

We investigated three main questions. First, do participants from different cultural groups rank animals with the same order preference? This question is interesting to explore in light of many findings which suggest that humans have particular affinity for certain animals (e.g., typically mammals) and less for others (e.g., typically reptiles) and to determine if these biases generalize across cultures. If culture impacts relative preferences for animals, we expect to discover significant variability in the patterns of liking across cultures.

Second, do people from diverse cultures have different attitudes with regard to familiarity, liking and endorsement for government protection toward threatened animals in Qatar? As there is little previous literature to predict whether or not cultural differences in familiarity, liking and endorsement for conservation will emerge, we investigated cultural questions from an exploratory stance. However, if culture plays a role, we expect Arabian Gulf participants to have a closer relationship to some of our species studied due to the role the species have in their culture.

Third, what is the likely mechanism that leads people to endorse government protection for threatened animals? Existing literature (e.g., Colléony et al., 2017; Echeverri et al., 2017) led us to predict that an individual’s willingness to endorse an animal for conservation will be mediated by animal liking, over and above familiarity. If this relationship can be demonstrated cross-culturally, it can help improve policy decisions and guide future research into successful interventions supporting animal conservation.

## Materials and Methods

This paper reports findings from a nation-wide survey in Qatar that was approved by the Carnegie Mellon University in Qatar (CMU-Q) local Institutional Review Board (Protocol #1603679-3). Data were collected between February 22 and April 9, 2021. Facebook and Instagram advertisements targeted Qatari residents and citizens who were 18 years of age or above. While scrolling through their Facebook/Instagram newsfeeds, potential participants were invited to “Volunteer to support research about awareness of Qatar’s natural heritage” and presented with a post containing the study flyer, information sheet, and a link to the online survey. All recruitment materials were provided in both Arabic and English. If participants wished to participate in the study, the link directed them to CMU-Q’s Qualtrics platform. Participants could then choose to take the survey in Arabic or in English. The survey consisted of three sections.

The first section included the Nature Relatedness Scale-6 (Nisbet and Zelenski, 2013) and modified questions about moral stances toward nature (Graham et al., 2009). The questions about moral stances explored participants’ views around protecting and treating animals fairly, agency in protecting animals, and the role of institutions in supporting protection. In the current set of analyses, these items were used as control variables and will be presented in a separate paper.

The second section asked participants to answer three questions about a diverse subset of threatened and indigenous species to Qatar and the Arabian Gulf. Participants were not told during the study that the animals were threatened. The five animals were chosen from the IUCN Red List for threatened and endangered species and because they are indigenous to Qatar, which means that Qatar’s government, citizens and residents play a critical role in conservation of these species:

- The dugong (*Dugong dugon)*, a close relative of the manatee and commonly referred to as a sea cow, is a relatively large marine mammal which is found in Qatar’s shallow coastal waters. The resident dugong population is the second largest population in the world after Australia (Preen, 2004; Marshall et al., 2018). Dugongs are threatened in Qatar mainly due to their slow reproduction, destruction of their coastal habitat, sea sport, commercial fishing, and pollution (Marsh and United Nations Environment Programme, 2002; Rabaoui et al., 2021). According to the IUCN, the dugong is vulnerable with decreasing status (Marsh and Sobtzick, 2019).
- The whale shark (*Rhincodon typus*), the world’s largest fish, appears between April and September about 90 km off Qatar’s coastline to feed on plankton and tuna eggs (Robinson et al., 2013). Observations indicate that the Arabian Gulf is home to one of the world’s largest gatherings of whale sharks (Bach and Al-Jaidah, 2012; Robinson et al., 2013) as it offers an abundance of food due to the unique marine characteristics of the region (Bach et al., 2014; Robinson et al., 2017). The whale shark’s life cycle and migration habits are poorly understood; therefore, its conservation requires global efforts. It is currently listed as endangered and decreasing (Pierce and Norman, 2016).
- The hawksbill sea turtle (*Eretmochelys imbricata*), a reptile, is the only sea turtle that nests in Qatar. Nesting occurs along Qatar’s eastern coastline between April and June (Chatting et al., 2018). About 200 sea turtles nest each year in three sites in Qatar, as well as other sites in the Arabian Gulf (Pilcher et al., 2014). The hawksbill sea turtle is listed as critically endangered with a decreasing status (Mortimer and Donnelly, 2008). In the Arabian Gulf, the hawksbill sea turtle faces threats due to rapid coastal expansion, widespread loss of marine habitat and climate change (Marshall et al., 2020).
- The saker falcon (*Falco cherrug)* is undergoing a rapid decline and is listed as endangered and decreasing by the IUCN (BirdLife International, 2021). The saker falcon is Qatar’s national bird and plays an important role in Qatar’s cultural heritage. Traditionally, the saker falcon had been used to hunt and is still used by locals for recreational hunting in all the Arabian Gulf states. The species faces threats due to trapping of birds for recreational use, pollution and habitat destruction.
- The spiny-tailed agama, locally known as Dhub (*Uromastyx aegyptia)*, is currently listed as vulnerable and decreasing by the IUCN (Wilms et al., 2012). It is a burrowing lizard that lives in loose colonies and feeds on low vegetation. This reptile is found on the Arabian Peninsula and throughout the Arabian Gulf countries such as parts of Egypt, Iraq, Iran and Jordan. It faces threats due to habitat loss from land reclamation, overgrazing, pollution and quarrying.

Participants were asked about each animal in succession in a randomized order. A color photograph of the animal was displayed, followed by these three questions:

1. Are you familiar with the <animal>?
2. How much, if at all, do you like the <animal>?
3. How much, if at all, do you think the government should prioritize protection of the <animal>?

Participants were given the following four-point Likert scale to respond to question 1: I do not recognize this animal; A little; Somewhat; A lot. Questions 2 and 3 were not presented if participants said they did not recognize the animal. For those respondents who indicated some degree of recognition, a four-point Likert scale with the following options was presented for questions 2 and 3: Not at all; A little; Somewhat; A lot.

In the final section of the survey, participants were asked their age, gender, nationality they most strongly identified with; highest level of education obtained and years living in Qatar.

A total of 3,418 social media users clicked on the link to our survey. Of these, 244 did not meet the inclusion criteria and 582 respondents did not complete the survey. Overall, a representative sample of 2,612 citizens and residents of Qatar completed the survey. From these, 218 participants chose not to divulge their nationality and are therefore not considered in the present analysis. Table 1 presents the demographics according to gender, age, education and years in Qatar of the remaining 2,394 respondents. Officially in Qatar, only 25% of the population is female; however, in our survey 47.8% of respondents were female. The official gender imbalance is skewed due to a large influx of foreign male laborers for construction projects. It is likely that the laborers did not participate in our survey, perhaps due to language barriers or disinterest. Participant ages ranged from 18 to over 81 years of age. The majority of the respondents were between the ages of 18–40 (84.5%) years of age. Those who participated were educated, with 78.3% of respondents indicating education beyond high school. Finally, reflecting the expatriate nature of many of Qatar’s residents, 28.1% of participants resided in Qatar for less than 5 years and a further 53.36% indicated living in Qatar between 5 and 20 years.

**Table 1:** Descriptive statistics for demographic characteristics.

	Characteristics	Number of respondents	Percentage
Gender	Female Male Prefer not to say	1,140 1,229 18	47.76 51.49 0.75
Age	18–22 23–30 31–40 41–50 51–60 61–70 71–80 81+	463 805 753 275 79 13 2 2	19.36 33.65 31.48 11.50 3.30 0.54 0.08 0.08
Education	Elementary school or less High school graduate College/Vocational/Associate’s degree Bachelor’s degree Master’s degree Professional degree (e.g., MD, JD, DD) PhD or other Doctoral degree	23 494 275 1,070 439 44 38	0.97 20.73 11.54 44.90 18.42 1.85 1.59
Years in Qatar	Less than 5 5–10 11–20 21–30 31–40 41–50 51–60 61–70 71+	665 649 614 290 89 43 13 0 4	28.09 27.42 25.94 12.25 3.76 1.82 0.55 0.00 0.17

Of the total 2,394 respondents used in our analysis, 2,387 answered the question about gender, 2,392 reported their age, 2,383 responded to the question about education, and 2,367 answered how long they had lived in Qatar.

Overall, respondents hailed from 96 unique nationalities (see Table 2). The most frequent nationality was Indian (31.7%), followed by Qatari (10.9%), Pakistani (7.8%) and Filipino/a (5.8%). This distribution is representative of the multi-cultural population in Qatar. For example, Indian nationals make up the largest percentage of any nationality living in Qatar at 21.9%, Pakistani nationals are estimated at 4.7%, Filipino/a nationalities at 7.4%, and Qataris at 10.5%. Note, Qatari nationals are a minority in Qatar (Snoj, 2019).

**Table 2:** Country names (respondent number) by regional group and associated group percentage of female respondents, median age, median education and median years in Qatar.

Group name (N)	Country (n)	% Female	Median age	Median education	Median years in Qatar
Arabian Gulf (273)	Qatar (261), United Arab Emirates (3), Saudi Arabia (4), Bahrain (1), Oman (3), Kuwait (1)	72	23–30	Bachelor’s Degree	21–30
MENA (505)	Algeria (25), Egypt (97), Iran (9), Iraq (5), Jordan (68), Lebanon (29), Libya (4), Morocco (16), Palestine (36), Sudan (95), Syria (47), Tunisia (42), Turkey (9), Yemen (23)	44	31–40	Bachelor’s Degree	11–20
Sub- Saharan Africa (125)	Burkina Faso (1), Cape Verde (1), Eritrea (6), Ethiopia (1), Ghana (5), Kenya (50), Mauritania (1), Mauritius (3), Mozambique (1), Nigeria (18), Somalia (9), South Africa (17), Tanzania (1), Uganda (10), Zimbabwe (1)	35	31–40	College/Vocational/Associate’s degree	5–10
South Asia (1,065)	Afghanistan (2), Bangladesh (60), India (758), Nepal (10), Pakistan (186), Sri Lanka (49)	36	23–30	Bachelor’s Degree	5–10
South East Asia (176)	Indonesia (18), Malaysia (14), Myanmar (1), Philippines (138), Singapore (5)	73	31–40	Bachelor’s Degree	5–10
WEIRD (172)	Australia (9), Austria (1), England (59), Canada (17), France (19), Germany (7), Ireland (8), Italy (7), Netherlands (1), New Zealand (2), Norway (1), Portugal (5), Scotland (4), Spain (6), Switzerland (1), Wales (2), USA (23)	63	31–40	Bachelor’s Degree	5–10
Mixed (78)	Albania (4), Argentina (1), Armenia (1), Azerbaijan (1), Belarus (1), Bosnia and Herzegovina (1), Brazil (6), Chile (1), China (3), Colombia (7), Costa Rica (2), Croatia (2), Estonia (1), Georgia (1), Greece (5), Hungary (1), Japan (2), Kazakhstan (3), Kyrgyzstan (4), Mexico (7), Peru (1), Poland (2), Romania (7), Russia (2), Serbia (1), South Korea (1), Suriname (1), Tonga (1), Tajikistan (1), Trinidad and Tobago (1), Ukraine (5), Venezuela (1)	79	31–40	Bachelor’s Degree	Less than 5

For data analysis, survey respondents were divided into six groups based on the nationality they most strongly identified with (Table 2): (a) Arabian Gulf (*n* = 273); (b) Middle East and North Africa (MENA, excluding Arabian Gulf; *n* = 505); (c) South Asia (*n* = 1,065); (d) South East Asia (S. East Asia, *n* = 176); (e) Sub-Saharan Africa (Africa, *n* = 125); and (f) WEIRD (*n* = 172). The WEIRD group was formed by combining nationalities from North America, Western Europe, Australia and New Zealand and has been previously described in the literature (Henrich et al., 2010; Klein et al., 2018). The remaining groups were created using the same regional criteria from the United Nations, Economic and Global Affairs, Statistics Division for use in the Sustainable Development Goals (SDG) indicators (United Nations Statistics Division, 2022). We divided the Arabian Gulf as a separate group from the MENA group in order to have more fine-grained analysis of the local population. We understand that there are variations in cultures within these large groupings, however based on other studies and on regional proximity, we feel that these are meaningful cultural groups. A group of “Mixed” participants was created (*n* = 78) which included participants from cultures and countries for which we did not have sufficient data to form regional groups, and for which we expected to see no systematic variation in animal preferences and attitudes based on the assumption that culture influences response patterns. Response rate by gender varied significantly across groups, with high female response rates in the Arabian Gulf, South East Asian, WEIRD and the Mixed groups. Interestingly, more males than females responded in the MENA and Sub-Saharan African groups. The WEIRD group tended to be older and more educated than the other groups, and the Arabian Gulf was the youngest group on average. MENA participants lived in Qatar for a similar number of years as their age, suggesting that many members of this group may have been born in Qatar. The other groups had spent significantly less time in the country, suggesting they were more transient expatriates.

Of the three main questions, first, we ask do participants from different cultural groups rank animals with the same order preference? To answer this question, we provide descriptive statistics for each cultural group’s preference rank. Besides, we conduct paired *t*-tests for ranking preferences to understand mean differences between the ranked animals by group.

Second, do people from diverse cultures have different attitudes toward threatened animals in Qatar? To address our second question, we implement a series of linear regression analyses where we consider three dependent variables: familiarity, liking, and endorsement of government protection, respectively. Our control variables include gender, age, education, years lived in Qatar, nature-relatedness scores (NR-6; Nisbet and Zelenski, 2013), and moral stances toward nature (based on Graham et al., 2009) as these variables could be correlated with the dependent variables and cultural backgrounds. Given the discrete nature of our dependent variables, we also run a series of ordered logistic regression analyses to ensure that our substantive conclusions were robust to different model choices.

Third, what is the likely mechanism that leads people to endorse government protection for threatened animals? To address our third question, we examine how participants’ familiarity with and liking of the threatened animals influence their endorsement of government protection for the animal. By answering this question, we aim to deepen our understanding of how the three different types of attitudes toward endangered animals are related. Our analysis for this question consists of two parts. First, we run three linear regression models to compare the effects of familiarity and liking on the endorsement of government protection. Second, we build on the linear regression analysis results to hypothesize a mechanism about how familiarity and liking influence the endorsement of government protection. To test our hypothesis, we use the potential outcomes approach to causal mediation analysis (Imai et al., 2010a,b) and the mediation R package (Tingley et al., 2014). This approach clarifies the identification assumptions required to estimate the mediation and the direct effects. Furthermore, it allows researchers to conduct a sensitivity analysis so that they can assess how much the estimated effect would be influenced by a possible violation of the assumptions.

## Results

### Ranking of Animal Preferences by Cultural Group

**Figure 1:**
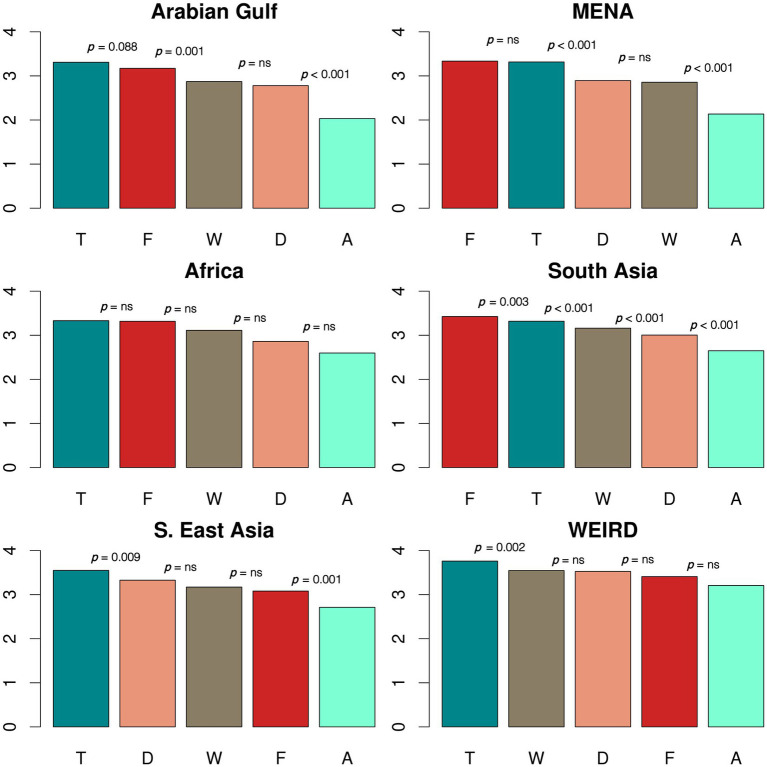
Ranking of Animal Preferences by Culture. D = Dugong. W=Whale shark. T = H.S. Turtle. F = S. Falcon, A = S.T. Agama. *p*: *p*-values from paired *t*-tests. ns: not significant at 0.05.

Figure 1 depicts the ranked order from most to least preferred animal across country groups. To summarize, each of the groups demonstrated clear animal preferences, and the order of animal preference follows a similar pattern across groups. The hawksbill sea turtle (T) is ranked or tied for first place as the preferred animal for each group, with the exception of South Asia, where it ranked second. The whale shark (W) and the dugong (D) are consistently grouped together in the rankings of each country group (i.e., they do not differ significantly from each other), except for the South Asian group. The spiny-tailed agama (A) was ranked significantly lowest by each cultural group, however note this difference was less pronounced in the WEIRD group. Only the saker falcon (F) shows meaningful rank differences across groups.

Figure 1 also depicts the paired *t*-tests’ *p*-values for the mean differences between the ranked animals by group. For instance, in the top left panel for the Arabian Gulf group the value of *p* = 0.088 reflects the mean difference between the hawksbill sea turtle and the saker falcon. Below, we interpret the paired *t*-test results briefly at the 0.05 significance level and detailed analysis is provided in Supplementary Figure S1.

The Arabian Gulf group’s preferences and the MENA group’s preferences are identical from a statistical point of view. The hawksbill sea turtle and the saker falcon are grouped together at the top of the ranking. The dugong was ranked significantly lower than the hawksbill sea turtle, but does not differ significantly from the whale shark. Finally, the spiny-tailed agama is liked the least.

Although none of the pairs for the African group’s mean difference tests reveal a significant difference at the 0.05 level, overall, it appears that the African group’s preferences are similar to the Arabian Gulf and MENA groups’ preferences. Similarly, the South Asian group’s overall pattern is more or less similar to the Arabian Gulf and MENA groups’ patterns, while the South Asian group’s rankings for the five animals are all significantly different.

Finally, the South East Asian group’s preferences and the WEIRD group’s preferences are similar to each other. Both groups like the hawksbill sea turtle significantly more than the other animals. The dugong, whale shark, and saker falcon are grouped together in second place. While the South East Asian group’s relative dislike for the spiny-tailed agama over the other animals is statistically significant, the WEIRD group’s tendency to rank the spiny-tailed agama lower than the saker falcon is short of statistical significance.

### Do Attitudes Toward Threatened Species Differ Across Cultures?

To address our second question, we implemented a series of linear regression analyses and a series of ordered logistic regression analyses to ensure that our substantive conclusions were robust to different model choices. We found that the substantive conclusions derived from the ordered logistic models were identical to the conclusions based on our linear regression models. Thus, we focus here on the results from our linear regression models without loss of generality; the ordered logistic estimation results can be found in Supplementary Figure S2.

**Figure 2:**
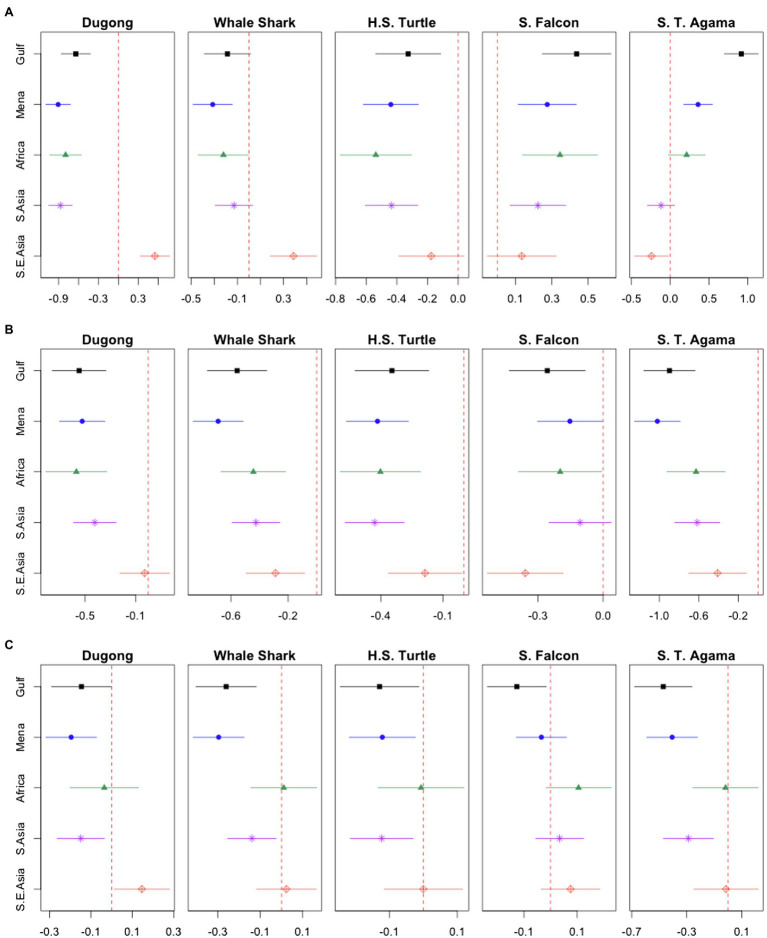
Ordinary least squares coefficients and 95% confidence intervals (CI): Comparing different nationalities. The red dotted line in each plot indicates the baseline WEIRD group. If a certain group’s 95% CI does not cross the red dotted line, it means that the group is significantly different from the WEIRD group at the 0.05 significance level. The “Mixed” group is omitted from the plot for the sake of simplicity though it is included in the analyses. Panel **(A)**: familiarity. Panel **(B)**: liking. Panel **(C)**: endorsement of government protection.

Figure 2 depicts the linear regression results for each animal across cultures for familiarity (Panel A), liking (Panel B) and endorsement of government protection (Panel C). For each of the models, the WEIRD group was used as a baseline (set to zero and depicted by the red dotted line) for comparison and effect sizes with 95% confidence intervals (CIs) are shown. If a group’s 95% CI does not cross the red dotted line, it means that the group is significantly different from the WEIRD group at the 0.05 significance level. We chose the WEIRD group as the baseline without loss of generality. Our substantive conclusions do not change when the baseline group varies. In order to make sense of the effect sizes, we can consider the following example. Panel A plots the country groups’ familiarity ratings for each animal. With respect to the dugong, the effect size value for the Arabian Gulf participants (depicted by the black square) is −0.64, which represents the difference between the Arabian Gulf group and the baseline WEIRD group for familiarity with the dugong. Thus, respondents from the Arabian Gulf are predicted to be less familiar with the dugong than the WEIRD group by 0.64, where the value of 1 means one level in the measurement used. This measurement was in response to the question “*Are you familiar with the < animal >?*” and used the Likert scale which ranged from 1 (“I do not recognize this animal”) to 4 (“a lot”). In contrast, if we examine the familiarity plot for the spiny-tailed agama (Panel A, far right), the Arabian Gulf respondents are predicted to be much more familiar with the spiny-tailed agama than the WEIRD group by about 1 Likert scale point (approximately 0.92).

Overall, the linear regression results demonstrate cultural differences for familiarity, liking and endorsement of government protection. Panel A reveals respondents from the Arabian Gulf and MENA regions, and to some degree Sub-Saharan Africa indicated increased familiarity for the saker falcon [Arabian Gulf: *t*(2,375) = 4.54, *p* < 0.000; MENA: *t*(2,375) = 3.37, *p* = 0.001; Africa: *t*(2,375) = 3.27, *p* = 0.001] and spiny-tailed agama [Arabian Gulf: *t*(2,372) = 8.27, *p* < 0.000; MENA: *t(*2,372) = 3.82, *p* < 0.000; Africa: *t*(2,372) = 1.74, *p* = 0.082] compared to the WEIRD group. All other groups indicated less familiarity with the dugong [Arabian Gulf: *t*(2,373) = −5.76, *p* < 0.000; MENA: *t*(2,373) = −9.63, *p* < 0.000; Africa: *t*(2,373) = −6.53, *p* < 0.000; South Asia: *t*(2,373) = −9.70, *p* < 0.000] and the hawksbill sea turtle [Arabian Gulf: *t*(2,372) = −3.02, *p* = 0.003; MENA: *t*(2,372) = −4.80, *p* < 0.000; Africa: *t*(2,372) = −4.54, *p* < 0.000; South Asia: *t*(2,372) = −4.98, *p* < 0.000] than WEIRD participants, except for South East Asians who indicated more familiarity with the dugong than the WEIRD group [*t*(2,373) = 4.94, *p* < 0.000]. For the whale shark, the MENA [*t*(2,373) = −3.63, *p* < 0.000] and Sub-Saharan Africa [*t*(2,373) = −1.97, *p* = 0.049] groups were significantly less familiar with the it than the WEIRD group, and the South East Asians were significantly more familiar with the whale shark than WEIRD participants [*t*(2,373) = 3.76, *p* < 0.000].

Panel B in Figure 2 depicts the linear regression results for each animal across cultures for liking. Generally, WEIRD participants showed a trend of increased animal liking compared to the other groups (*p*-values range from 0.000 to 0.044), except for the dugong for which the South East Asian and the WEIRD participants do not differ significantly [*t*(1,579) = 0.278, *p* = 0.781] and the saker falcon for which the South Asian and the WEIRD groups do not differ [*t*(2,257) =1.47, *p* = 0.143]. Examining the 95% CIs, it is clear that the WEIRD group behaves exceptionally: most of the other groups do not differ significantly from one another with regards to animal liking. It is striking that the Arabian Gulf group does not like the saker falcon [*t*(2,257) = −2.89, *p* = 0.004] or spiny-tailed agama [*t*(1,549) = −6.81, *p* < 0.000] more than WEIRD participants, considering the high familiarity (see Panel A) and the cultural significance of these animals.

Finally, Panel C depicts the linear regression results for each animal across cultures for endorsement of government protection, our proxy for the WTP procedure used in other studies. The Arabian Gulf participants endorsed all of the animals less for protection than WEIRD participants [dugong: *t*(1,573) = −1.98, *p* = 0.048; whale shark: *t*(2,216) = −3.60, *p* = 0.000; hawksbill sea turtle: *t*(2,127) = −2.18, *p* = 0.029; saker falcon: *t*(2,246) = −2.25, *p* = 0.025; spiny-tailed agama: *t*(1,540) = −4.44, *p* < 0.000]. MENA and South Asian participants behaved similarly, endorsing all animals, except the saker falcon, less for protection than WEIRD participants [*MENA*: dugong: *t*(1,573) = −3.14, *p* = 0.002; whale shark: *t*(2,216) = −4.83, *p* < 0.000; hawksbill sea turtle: *t*(2,127) = −2.43, *p* = 0.01; spiny-tailed agama: *t*(1,540) = −4.32, *p* < 0.000. *South Asia*: dugong: *t*(1,573) = −2.55, *p* = 0.011; whale shark: *t*(2,216) = −2.39, *p* = 0.017; hawksbill sea turtle: *t*(2,127) = −2.60, *p* = 0.009; spiny-tailed agama: *t*(1,540) = −3.11, *p* = 0.002]. South East Asian and Sub-Saharan African participants did not differ from the WEIRD group, except in the case of the dugong, where South East Asians were more likely to endorse it for protection [*t*(1,573) = 2.15, *p* = 0.032].

### Which Mechanism Might Lead to Endorsement of Government Protection for Threatened Animals?

To address our third question, we examined how participants’ familiarity with and liking of the threatened animals influence their endorsement of government protection for the animal. By answering this question, we aim to deepen our understanding of how the three different types of attitudes toward endangered animals are related.

**Figure 3:**
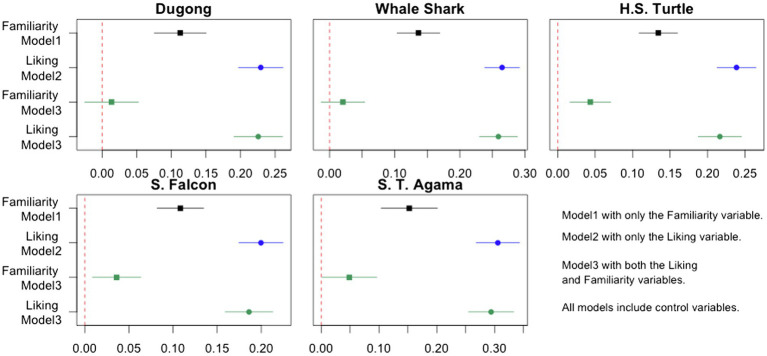
Ordinary least squares coefficients and 95% confidence intervals: Comparing the effects of familiarity and liking on the endorsement of government protection.

First, we ran three linear regression models to compare the effects of familiarity and liking on the endorsement of government protection. Figure 3 displays our regression analysis results. Each of the five plots displays the ordinary least squares coefficients and 95% CIs that come from three different linear regression models. In Model 1 and Model 2, we investigate the relationship between familiarity and endorsement and the relationship between liking and endorsement, respectively. Model 3 includes both familiarity and liking as predictors for the endorsement of government protection.^1^

In order to introduce Figure 3, let us take the example of the dugong: the black square and the black line in the plot for the dugong indicate the estimated effect of familiarity with the dugong on the endorsement of government protection for the animal and its 95% CIs. These are derived from Model 1 where only the variable for familiarity, along with the control variables, are included. On the other hand, the green square and the green line in the same plot represent Model 3’s familiarity variable.

Most notably, the results suggest that the effect of familiarity diminishes significantly after accounting for liking, whereas the effect of liking remains virtually the same after accounting for familiarity. Continuing to use the dugong example, the estimated effect of familiarity is about 0.11, and it is statistically distinguishable from zero at the 0.05 level according to Model 1 [*t*(1,530) = 5.92, *p <* 0.000]. However, the estimated effect shrinks to about 0.014, and it is no longer different from zero at the 0.05 level according to Model 3 [*t*(1,524) = 0.69, *p* = 0.490]. Similar patterns appear in the other animal cases such that the effect of familiarity weakens significantly after the variable for liking is also considered. In contrast, the estimated effect of liking obtained from Model 2 does not significantly decline after controlling for familiarity in Model 3. For example, the blue circle in the dugong case indicates that the estimated effect of liking is about 0.229 [*t*(1,527) = 14.00, *p* < 0.000] in Model 2. The green circle in the same plot reveals that the estimated effect of liking is about 0.225 [*t*(1,524) = 12.64, *p* < 0.000] after the variable for familiarity is included in Model 3.

These results allow us to better understand how the three types of attitudes toward threatened animals are related. First, the extent to which participants are familiar with a threatened animal does not uniquely explain their endorsement of government protection for the animal. Rather, the amount of variance in the endorsement of government protection explained by familiarity mostly overlaps with what liking explains. Second, although familiarity and liking are correlated and jointly explain the endorsement of government protection, liking explains a large amount of unique variance in the endorsement of government protection that familiarity cannot explain.

We continued to build on the above results to delve further into the relationship between the three types of attitudes toward threatened animals. Our finding that familiarity does not uniquely explain participants’ endorsement of government protection while liking does might be due to the mediating role of liking between familiarity and the endorsement of government protection. In other words, the effect of familiarity with an animal on the endorsement of government protection might exert mostly *via* how much they like the animal.

**Figure 4:**
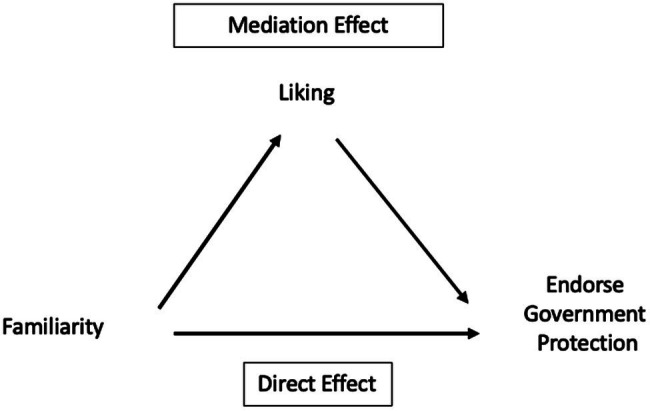
Model. The total effect of familiarity on the extent to which people endorse government protection is decomposed into two causal pathways. The top pathway reflects a mechanism through which the effect of the extent to which people are familiar with the threatened animal is mediated by how much they like the animal. The bottom direct pathway between familiarity and endorsement represents alternative mechanisms not intervened by how much they like the animal.

In Figure 4, the mediation effect graphically illustrates our hypothesis, which suggests that the extent to which participants like the animal mediates the effect of familiarity on the endorsement of government protection. This hypothetical mechanism suggests that the more familiar with the animal people are, the more they like the animal, and once they like the animal, they tend to endorse government protection for the animal.

The bottom pathway in Figure 4 captures all other mechanisms not intervened by our hypothetical mechanism. We call this pathway the direct effect of familiarity on the endorsement of government protection. The total effect of familiarity on the endorsement of government protection is the sum of the mediation effect and the direct effect.

We build on the potential outcomes approach to causal mediation analysis (Imai et al., 2010a,b) to test our hypothesis. We made two assumptions to identify the mediation and direct effects in our analysis, following the sequential ignorability assumption proposed by Imai et al. (2010b). First, we assume familiarity is independent of potential outcomes for endorsement of government protection and liking, after controlling for pretreatment covariates. Since the extent to which people are familiar with an animal is based on their knowledge, we are less concerned about the possibility that people’s subjective liking or endorsement of government protection affects familiarity. Nevertheless, individual characteristics associated with liking could be correlated with familiarity. Hence, we control for demographic characteristics, nature-relatedness, and moral stances toward nature as pretreatment covariates to minimize contamination from individual characteristics.

The second assumption is that the extent to which people like a threatened animal is independent of potential outcomes for endorsement of government protection given the observed pretreatment covariates and the observed values for familiarity. Similar to the first assumption, this assumption would be violated if individual characteristics associated with endorsing government protection are correlated with familiarity or liking. Thus, we included the same pretreatment covariates as in the previous analyses to minimize a possible violation of the assumption. Additionally, we implemented a sensitivity analysis to ensure that a possible violation of the first and second assumptions would not significantly change our analysis results. The results of the sensitivity analysis can be found in Supplementary Figure S3.

We used the mediation R package (Tingley et al., 2014). The mediation package performs a mediation analysis based on two steps. The first step fits two regression models. To begin with, we fit the model for the mediating variable--liking. The right-hand side of this model includes the treatment variable--familiarity and pretreatment covariates. We also fit the model for the outcome variable--endorsement, in which the right-hand side variables include the same variables as the first model and additionally the mediating variable. The pretreatment covariates used in each of these two models are demographic characteristics (age, gender, and education), the composite measure of nature-relatedness, and the seven variables for moral stances toward nature. Thereafter, the second step computes the mediation effect and the direct effect using simulated potential values that are generated from the two regression models.

**Figure 5:**
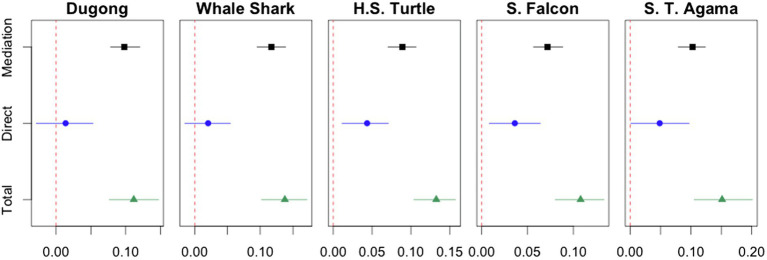
Mediation analysis results: estimated mediation, direct, and total effects and 95% nonparametric bootstrap confidence intervals. Red dotted lines at 0 indicate a null effect.

The results are displayed in Figure 5. Each point in Figure 5 is an estimated effect of a one-level increase in familiarity on the likelihood of endorsing government protection. Each horizontal line indicates uncertainty about the estimated effect at the traditional 0.05 level. The red dotted lines indicate a null effect.

The estimated mediation effect represented by the black square point in each plot is positive and statistically distinguishable from zero in every case (*p*s < 0.000).^2^ For example, the black square point in the left-most plot indicates that a one-level increase in participants’ familiarity with the dugong is predicted to increase the level of endorsing protection for the animal by about 0.1 *by making people like the dugong*. All other mechanisms not intervened by liking, which correspond to the direct effect, do not have a significant impact on the likelihood of endorsing government protection for the dugong (*p* = 0.44).

A similar pattern appears in every case as the five plots in Figure 5 show. Although the direct effect is statistically significant at the 0.05 level in the cases of the hawksbill sea turtle (*p* = 0.004) and the saker falcon (*p* = 0.02), it contributes to the total effect far less than the mediation effect. To be specific, the mediation effect is predicted to contribute to about 0.65–0.88 of the total effect in every case.

These results support our hypothesis that the extent to which people like a threatened animal mediates the link between people’s familiarity with the animal and their endorsement of government protection. We also find that these results remain constant across different cultural groups. Based on our findings, we suggest that there exists a cross-cultural mechanism through which people’s familiarity with an animal at risk tends to make them like the animal, and their subjective liking, in turn, increases the likelihood of their endorsing government protection for the animal.

## Discussion

The present study provides the first comparison of attitudes toward threatened animals and conservation across a wide range of cultural groups, including understudied populations. The goal of this research was threefold: to assess the ranking of animals across diverse cultural groups; to examine the ways in which responses to questions about familiarity, liking and endorsement for government protection toward indigenous animals in the Arabian Gulf might vary across diverse cultural groups; and to explore potential mechanisms leading people to endorse government protection for threatened animals. As the first study about this topic to address a diverse sample from a single geographic location, we shed light on how the attitudes of people toward animals compare across cultures. Our data reveal both strikingly similar shared attitudes toward animals across the diverse cultures studied and some intriguing cultural variation.

Previous findings (e.g., Martín-López et al., 2007) have demonstrated that people living closer in geographic distance to animals are more aware of and express more concern for animals near to them. In our study, all participants lived in the same small peninsular state of Qatar, and thus were similarly geographically located. A primary difference between participants in this study is in cultural backgrounds. In our analyses, WEIRD participants were compared with participants from the Arabian Gulf, Middle East and North African (MENA), Sub-Saharan Africa, South East Asia, and South Asia.

### Similarities Across Cultures

One of the most important results from our study was the influential role of a person’s subjective liking of animals on their opinions about the need for animal conservation. Despite the fact that the participants in our study were interested in animals and conservation (as suggested by their self-selection without incentive to participate, and overall high levels of liking for animals), their responses exhibited significant variability in attitudes toward animals. The general agreement across cultures in ranking order is striking: Across every culture, the hawksbill sea turtle was highly ranked and the spiny-tailed agama ranked consistently lowest. It is interesting that the dugong did not rank higher, as mammals are typically ranked the most charismatic species, at least amongst Westerners, although most species ranked as charismatic are terrestrial (Albert et al., 2018; Courchamp et al., 2018). Although reptiles are generally considered to be non-charismatic species (e.g., Tisdell et al., 2006), turtles have been shown to be an exception in other studies and cultures (e.g., Senko et al., 2011; Borgi and Cirulli, 2015). Other studies have found that a fear of reptiles appears across cultures (Arrindell, 2000; Ceríaco, 2012; Pandey et al., 2016; Pinheiro et al., 2016) and from a young age (e.g., Prokop and Fančovičová, 2013), suggesting a culturally universal dislike for most reptiles. The spiny-tailed agama is a fairly typical reptile, but the hawksbill sea turtle is arguably atypical: It is graceful, non-threatening, and its survival is clearly jeopardized by human activities. It would seem our results support the finding that the hawksbill sea turtle is a strikingly charismatic marine reptile that could potentially serve as a universally appealing flagship species. In Qatar, the high ranking of the hawksbill sea turtle could be also be influenced by recent efforts to raise awareness. For example, the turtles have appeared periodically in the local news and the Qatari government closes a popular beach in the summer for nesting. It is possible that these conservation activities have influenced people’s perceptions and attitudes to some degree. To address these possibilities, future research is needed to understand how marketing campaigns and conservation efforts in Qatar impact attitudes, perceptions and behaviors of residents.

Additionally, as nuances in charisma between and within species have been reported in the literature, it would be incorrect to assume that all mammals are charismatic and all reptiles are not (e.g., Stokes, 2007; Prokop and Fančovičová, 2013). The dugong and whale shark appeared closely together in their rankings across all groups, perhaps because of their large size and similar appearance (Kellert, 1993; Metrick and Weitzman, 1996; Ward et al., 1998; Tisdell et al., 2006). Previous research would predict higher rankings for these animals due to their large size; however, they placed below the hawksbill sea turtle and, for some cultures in our study, also below the saker falcon. It is possible that these large marine animals evoked some degree of fear based on historical ideals of marine animals (see Mazzoldi et al., 2019 for discussion).

Previous studies have highlighted the importance of phylogenetic closeness in influencing liking and conservation of animal species (e.g., Colléony et al., 2017; Prokop et al., 2021). The hawksbill sea turtle was consistently ranked highly, and this species is more phylogenetically distant from humans than other species (e.g., the dugong is a mammal). Our findings suggest that phylogenetic closeness is not a major determinant of attitudes, or is at least one of multiple factors influencing attitudes (see Fančovičová et al., 2021 for a similar discussion). Altogether, our data demonstrate how people, irrespective of culture, readily rank animals according to their preferences and that, with some nuances, there is remarkable similarity in animal rankings across the cultural groups. Both outcomes are interesting and seem to be largely independent of culture. We will discuss the saker falcon ranking and the few group differences related to ranking in more detail below.

Our most intriguing result demonstrates how participants’ willingness to endorse animals for government protection was based on their personal preferences for animals, regardless of culture. These findings are in line with previous research using WTP paradigms, which also found that participants’ endorsements in the form of donations were predicted by animal liking and not by other factors such as threatened status of the animal or knowledge of animals in the general population (Colléony et al., 2017; Echeverri et al., 2017) though this may not hold true for specialists. Previously, Tisdell et al. (2005) reported likeability as the main predictor of public support for wild animals in Australia, whereas the importance of likeability, which may be related to empathy, aesthetics or other factors particular to human preferences have also been noted in previous research (DeKay and McClelland, 1996; Metrick and Weitzman, 1996; Gunnthorsdottir, 2001). Our data suggest that decision making about animal conservation worldwide, irrespective of culture, will be strongly influenced by subjective liking. This finding is important because it corroborates previous research which has demonstrated how preferences have influenced policy making (Coursey, 1994; Metrick and Weitzman, 1996; Czech et al., 1998; Knegtering et al., 2002; Mahoney, 2009).

It may be that decisions about conservation are being influenced by what Kahneman (2011) coined as System 1, rather than System 2. System 1 is described as our default decision making system, which relies on biases, heuristics and emotions to make quick and efficient decisions, whereas System 2 is slower, methodical, critical and aware. Consideration of our results in light of existing literature on decision making suggests that policy makers could benefit from understanding how their own personal biases toward conservation issues might influence their decisions and how they can leverage thoughtful and unbiased approaches to the implementation and design of related policies, including communication with the public. Further interventions could include explicitly teaching children this thoughtful approach in schools and encouraging the public to think about the consequences of treating animals unequally (i.e., the impact of imbalances in biodiversity on ecosystems).

Finally, the similarities described here across cultures (i.e., the finding that people are influenced by their subjective liking when making decisions about endorsement for government protection, as well as the similarity in animal ranking across cultures) suggest the presence of cultural universals. Cultural universals are interesting because their presence indicates that some cognitive processes operate largely outside the influence of the environment (Norenzayan and Heine, 2005). The power in identifying cultural universals is in the generalizability of psychological findings across cultures. In the case of attitudes toward animals, our data suggest that the role that liking plays in conservation efforts should be taken into consideration when designing conservation campaigns globally.

### Differences Between Cultures

Our analyses revealed that overall, the different cultural groups expressed varying degrees of familiarity toward the animals. Not surprisingly, the saker falcon and the spiny-tailed agama were most familiar to participants from Arabian Gulf, MENA and Sub-Saharan African countries. Interestingly, although all animals are native to the Arabian Gulf, participants from this region were less familiar with the dugong, spiny-tailed agama and hawksbill sea turtle than the WEIRD and/or South East Asian groups. Our mediation analysis reveals that familiarity was not a strong predictor of endorsement of government protection. It would seem that familiarity is a means by which someone might come to know of an animal, and may drive liking to some degree, but is not as important as liking in driving endorsement of government protection of animals.

Although participants generally liked the animals (with the exception of the spiny-tailed agama), there were group differences in the degree of liking. WEIRD and South East Asian participants consistently indicated more positive attitudes toward animals than other groups, even when they were less familiar with the species (i.e., saker falcon and spiny-tailed agama). It is striking that Arabian Gulf participants liked all animals less than WEIRD participants, even though these animals are indigenous to their home region and in the case of the saker falcon and the spiny-tailed agama, play significant cultural roles. The attitudes held by respondents from the Arabian Gulf were consistent with the attitudes of the overall MENA group, and to a large degree respondents from Sub-Saharan Africa as well, thus suggesting that lower levels of animal liking might be more common in these regions.

Interestingly, the saker falcon was the only animal which changed rank dramatically between cultural groups. It is not surprising that animal preferences would be influenced by culture, but it is surprising that only the saker falcon shows strong variation. A possible explanation for why it ranked higher in the Arabian Gulf, MENA, Sub-Saharan African and South Asian contexts could be that it fills a special role or ideal for those cultural groups. For example, in Qatar, the saker falcon was used traditionally for hunting and is still used by locals in recreational hunting today. On the other hand, another animal with cultural significance in Qatar and the MENA region, the spiny-tailed agama, ranked lowest; it is an animal that locals encountered frequently in the desert and was a source of food in the past. While the saker falcon still serves as an important cultural symbol, the spiny-tailed agama does not, so perhaps cultural relevance does not influence its ranking.

### Conclusion

The majority of psychological research studies have been conducted with WEIRD populations; without further input from other global perspectives, psychological theory is limited. Research on cross-cultural perspectives of pressing global concerns can provide much needed guidance and advice for policy makers. Our research sheds light on global perspectives by comparing participants from WEIRD countries with previously understudied or unstudied populations from the Arabian Gulf, MENA, Sub-Saharan Africa, South Asia and South East Asia on their attitudes toward animals and conservation. All participants lived in the same geographic location, Qatar, and were tested using the same instrument within a short period of time. The animals under investigation are all indigenous to the Arabian Gulf and are all threatened species according to the IUCN red list.

Overall, participants generally indicated they liked most animals, and for the most part, ranked animals in a similar order across cultures. However, reported levels of liking were higher for WEIRD and South East Asians than for other groups. Although participants were likely to be animal enthusiasts, they all showed clear preferences for certain animals over others; for example, ranking the hawksbill sea turtle as consistently more liked and the spiny-tailed agama as less liked than the other animals. Across groups, liking emerged as the primary predictor for endorsement of government protection over and above familiarity, which independently explained very little significant variance. Future research should explore liking as a mechanism for improving attitudes toward animals and conservation decisions. Previous studies with adults have demonstrated improved liking toward animals when education has both empathy and biological literacy components (Candea, 2010; Root-Bernstein et al., 2013; de Pinho et al., 2014). Studies with school children further demonstrated the power of education on improving attitudes toward typically disliked animals (Ballouard et al., 2012; Randler et al., 2012; Collado et al., 2021). Indeed, a great deal of research points to the critical role that early education (both formally and informally) and experiences in natural settings have on shaping attitudes toward animals, and that these experiences shape future thinking (Dunlap, 1989; Soga and Gaston, 2016; Whitburn et al., 2020). Tactics suggested by this literature can be applied to improve conversation attitudes toward the spiny-tailed agama. Developing a program to raise awareness of the spiny-tailed agama through providing opportunities for Qatari residents to interact with and even handle these animals in a controlled environment may reduce negative attitudes and increase liking and conservation endorsement for these important creatures (Prokop et al., 2009; Randler et al., 2012).

In the Arabian Gulf, the local populations have the most control over conservation in the region and yet our research demonstrates attitudes toward animals and conservation amongst this group are the least favorable of the groups surveyed. Thus, it seems vital to improve animal liking in the Arabian Gulf to support conservation of these indigenous species. Qatar has well-defined goals to support biodiversity within its borders (Qatar General Secretariat for Development Planning, 2011; Qatar Ministry of Environment, 2014), and the current study suggests that increasing liking will support attainment of these goals. Because our data suggest that the role of liking in conservation is universal across cultures, it is theoretically possible to generalize from lessons learned in other cultures on improving liking and conservation to shape education efforts.

## Data Availability Statement

The datasets and R code used in this study can be found in Harvard Dataverse: https://dataverse.harvard.edu/dataset.xhtml?persistentId=doi:10.7910/DVN/NGWFFU.

## Ethics Statement

The studies involving human participants were reviewed and approved by Carnegie Mellon University in Qatar (CMU-Q) local Institutional Review Board. Written informed consent for participation was not required for this study in accordance with the national legislation and the institutional requirements.

## Author Contributions

JB, LB, RA-H, SA-H, AA-K, and AA-N conceptualized and designed the study, and collected the data. JB and TP conducted statistical analyses. JB, LB, and TP wrote and edited all drafts of the article and approved the submitted version. All authors contributed to the article and approved the submitted version.

## Funding

This research was made possible by UREP grant #UREP26-107-5-024 from the Qatar National Research Fund (a member of Qatar Foundation). The statements made herein are solely the responsibility of the authors.

## Conflict of Interest

The authors declare that the research was conducted in the absence of any commercial or financial relationships that could be construed as a potential conflict of interest.

## Publisher’s Note

All claims expressed in this article are solely those of the authors and do not necessarily represent those of their affiliated organizations, or those of the publisher, the editors and the reviewers. Any product that may be evaluated in this article, or claim that may be made by its manufacturer, is not guaranteed or endorsed by the publisher.

## Footnotes

^1^The three models’ control variables are consistent with the control variables used in the previous subsection. Full regression analysis results are available in Supplementary Tables S1-S5.

^2^The values of *p* were computed using nonparametric bootstrap confidence intervals by the mediation package.
