# Supplementary material for: Cross-Cultural Awareness and Attitudes Toward Threatened Animal Species
Source: Front Psychol. 2022 May 31;13:898503. doi: 10.3389/fpsyg.2022.898503 (PMC9194822; doi:10.3389/fpsyg.2022.898503)
Supplement: Supplementary file 1 [file Image_1.pdf]

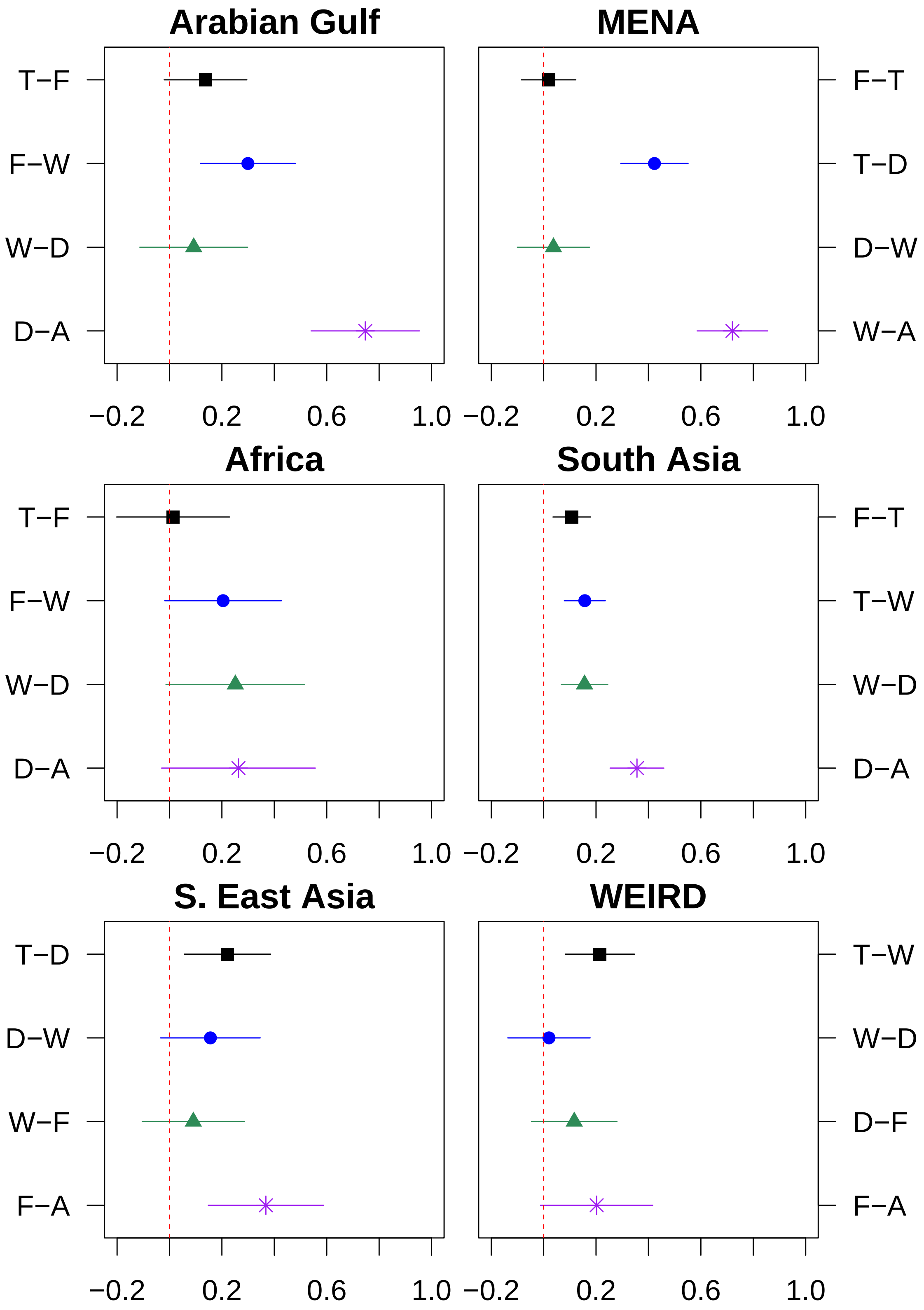

Figure S1. Paired *t*-test Results with 95% Confidence Intervals. [*Note*: Figure 1 of the main text depicts the cultural groups' animal preferences. Figure S1 provides paired *t*-test results for each adjacent pair of animals in the ranking presented in Figure 1. For example, in the top-left panel for Arabian Gulf, T-F refers to the difference between the hawksbill sea turtle and the saker falcon, and it is presented at the top because the two animals are ranked first and second in the Arabian Gulf group. The red dotted lines at 0 indicate no difference. If the 95% confidence interval crosses the red dotted line, it means no statistical difference at the 0.05 significance level.]
