# Supplementary material for: Cross-Cultural Awareness and Attitudes Toward Threatened Animal Species
Source: Front Psychol. 2022 May 31;13:898503. doi: 10.3389/fpsyg.2022.898503 (PMC9194822; doi:10.3389/fpsyg.2022.898503)
Supplement: Supplementary file 2 [file Image_2.pdf]

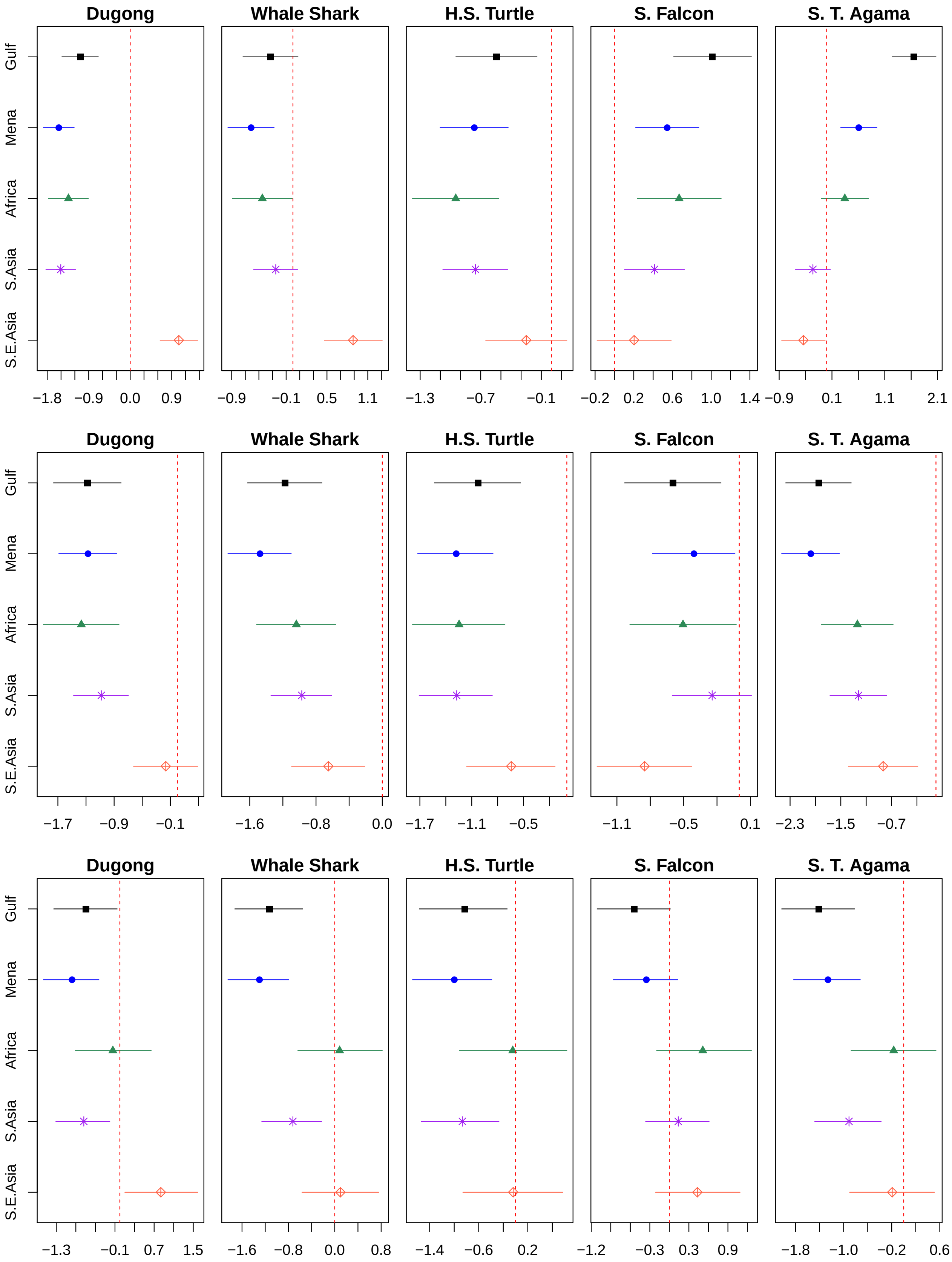

Figure S2. Ordered Logistic Regression Coefficients and 95% Confidence Intervals. [*Note*: The results displayed in this figure are virtually identical to the results in Figure 2. That is, our results in the main text are robust to different model choices. The red dotted line in each plot indicates the baseline WEIRD group. If a certain group's 95% confidence interval does not cross the red dotted line, it means that the group is significantly different from the WEIRD group at the 0.05 significance level. The "Mixed" group is omitted from the plot for the sake of simplicity though it is included in the analyses.]
