# Supplementary material for: Cross-Cultural Awareness and Attitudes Toward Threatened Animal Species
Source: Front Psychol. 2022 May 31;13:898503. doi: 10.3389/fpsyg.2022.898503 (PMC9194822; doi:10.3389/fpsyg.2022.898503)
Supplement: Supplementary file 3 [file Image_3.pdf]

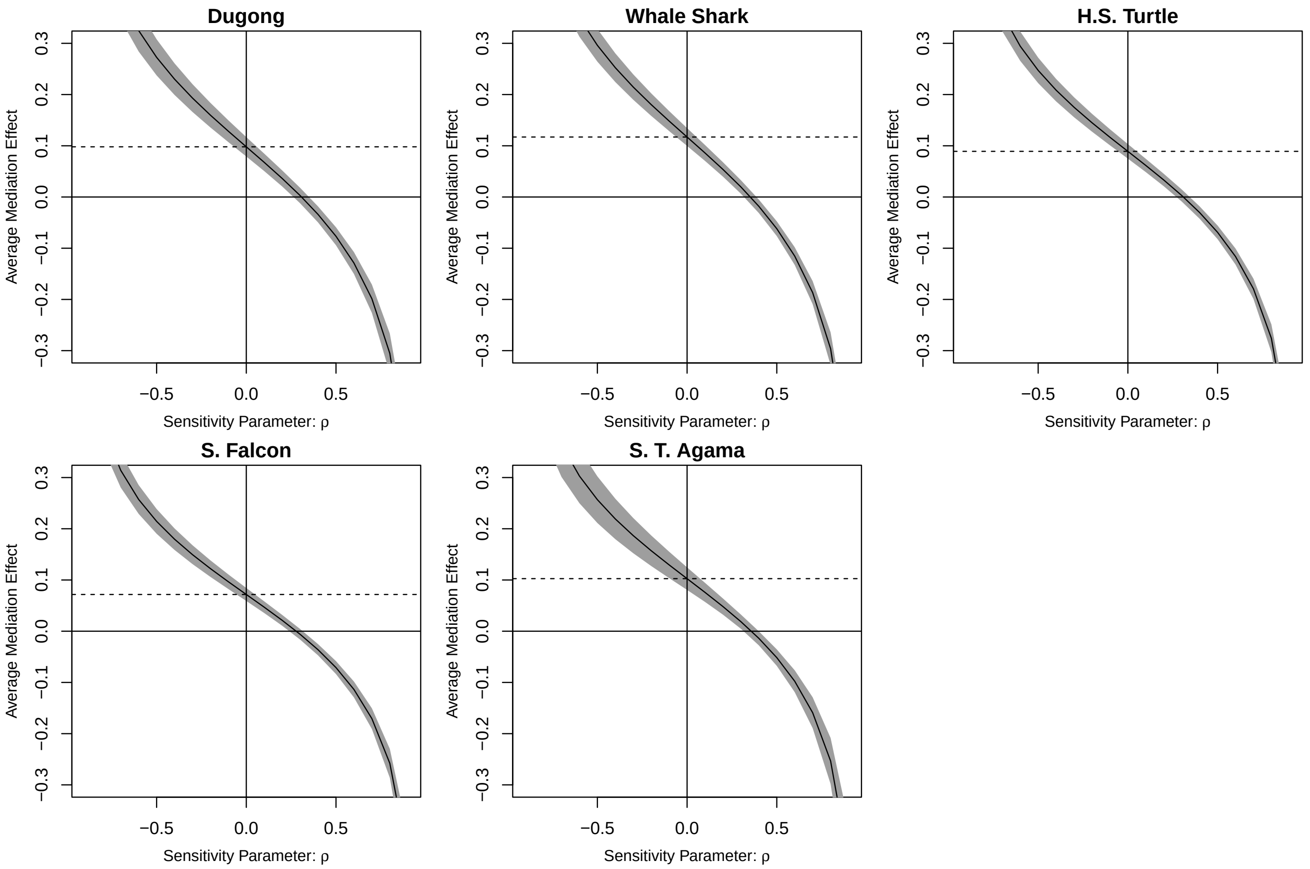

Figure S3. Sensitivity Analysis. [*Note*: The mediation effects displayed in the main text's Figure 5 represent the estimated average mediation effects under the sequential ignorability assumption, $\rho = 0$, where ρ denotes the correlation between the errors in the first and the second stage of the regression model. Our sensitivity analysis is to relax this sequential ignorability assumption to see how much the estimated average mediation effects would be sensitive to ρ's departures from zero. We used the mediation R package to execute sensitivity analysis. Each of the five plots in the figure shows the estimated average mediation effects that vary across different values of ρ. First of all, we see that when $\rho = 0$, the estimated average mediation effect in every case is statistically distinguishable from zero, which is consistent with the results reported in the main text. Besides, we can estimate how much ρ must deviate from zero to make the estimated average mediation effect indistinguishable from zero. For example, in the dugong case, the estimated average mediation effect would have been null, instead of a positive value, if ρ was in fact 0.3 rather than zero. Similar sizes of ρ would make the estimated average mediation effects null in the other plots. Overall, these sizes of ρ seem large enough to suggest that just a little amount deviation from zero would not debunk our conclusion. In other words, our sensitivity analysis suggests that the results presented in Figure 5 of the main text are fairly robust to a potential violation of the sequential ignorability assumption.]
