# Supplementary material for: Cross-Cultural Awareness and Attitudes Toward Threatened Animal Species
Source: Front Psychol. 2022 May 31;13:898503. doi: 10.3389/fpsyg.2022.898503 (PMC9194822; doi:10.3389/fpsyg.2022.898503)
Supplement: Supplementary file 7 [file Table_4.pdf]

Table S4. Linear Regression Models of Endorsement for Government Protection: S. Falcon

	Model 1	Model 2	Model 3
(Intercept)	2.01*	1.99*	1.91*
	(0.24)	(0.24)	(0.24)
familiarFalcon	0.11*		0.04*
	(0.01)		(0.01)
likeFalcon		0.20*	0.19*
		(0.01)	(0.01)
nr6	0.12*	0.04	0.04
	(0.04)	(0.03)	(0.03)
protectAnimals	0.07	0.03	0.03
	(0.04)	(0.04)	(0.04)
treatFairly	−0.03*	−0.02	−0.02
	(0.01)	(0.01)	(0.01)
reportIllegal	0.02	0.02	0.02
	(0.02)	(0.02)	(0.02)
obeyAnimalLaw	0.14*	0.13*	0.13*
	(0.05)	(0.05)	(0.05)
disgustedGarbage	0.18*	0.17*	0.17*
	(0.06)	(0.06)	(0.06)
godWill	0.01	0.01	0.01
	(0.02)	(0.02)	(0.02)
ableProtect	−0.02	−0.03	−0.03
	(0.02)	(0.02)	(0.02)
age	0.02	0.01	0.01
	(0.01)	(0.01)	(0.01)
education	−0.00	0.00	0.00
	(0.01)	(0.01)	(0.01)
female	0.06*	0.09*	0.09*
	(0.02)	(0.02)	(0.02)
howLongQatar	−0.01	−0.00	−0.00
	(0.01)	(0.01)	(0.01)
nationGulf	−0.19*	−0.10	−0.12*
	(0.06)	(0.05)	(0.05)
nationMena	−0.08	−0.02	−0.04
	(0.05)	(0.05)	(0.05)
nationAfrica	0.05	0.12*	0.11
	(0.06)	(0.06)	(0.06)
nationEastAsia	0.06	0.13*	0.12*
	(0.05)	(0.05)	(0.05)
nationSouthAsia	−0.01	0.03	0.02
	(0.04)	(0.04)	(0.04)
nationMixed	−0.00	0.00	−0.00
	(0.07)	(0.07)	(0.07)
N	2208	2200	2200
adj. R^2	0.07	0.14	0.14

Standard errors in parentheses.

* indicates significance at $p < 0.05$.

[*Note*: Every model is a linear regression model of endorsement for government protection. The information about the dependent variable and the "familiar<animal>" and "like<animal>" variables is available on Pages 4-5 of the main text. The "nr6" variable is a composite measure for nature-relatedness (Nisbett and Zelensky, 2013). It takes the mean of the responses to six different questions designed to measure nature-relatedness: "My ideal vacation spot would be a remote, wilderness area," "I always think about how my actions affect the environment," "My connection to nature and the environment is a part of my spirituality and/or religion," "I take notice of wildlife wherever I am," "My relationship to nature is an important part of who I am," and "I feel very connected to all living things and the earth." Each of these six questions has three response options, 1, 2, and 3. A higher value in the response indicates a higher level of nature-relatedness. The next seven variables are modified from Graham et al. (2009) and all responses are scaled 1, 2, and 3 with higher responses indicating agreement. The "protectAnimals" variable question is: "It is the moral responsibility of humans to protect animals living in their natural habitats from harm." The "treatFairly" variable question is: "Humans treat animals living in their natural habitats fairly." The "reportIllegal" variable question is: "If I knew that a member of my family had illegally killed an endangered species, I would report them to the authorities." The "obeyAnimalLaw" variable question is: "I would obey any law the government made to protect animals in their natural habitats." The "disgustedGarbage" variable question is: "I feel disgusted when I see garbage in the desert and the sea." The "godWill" variable question is: "It is God's will that we protect animals in their natural habitat." The "ableProtect" variable question is: "I am able to meaningfully contribute to the protection of animals in their natural habitat." The demographic variables are given as levels as described in the main text. Each of the national variables is an indicator variable.]

REFERENCES

Graham, J., Haidt, J., and Nosek, B. (2009). Liberals and conservatives rely on different sets of moral foundations. J. Pers. Soc. Psychol. 96, 1029–1046. doi: 10.1037/a0015141

Nisbet, E. K., and Zelenski, J. M. (2013). The NR-6: a new brief measure of nature relatedness. Front. Psychol. 4:813. doi: 10.3389/fpsyg.2013.00813
